# Supplementary material for: Context dependent isoform specific PI3K inhibition confers drug resistance in hepatocellular carcinoma cells
Source: BMC Cancer. 2022 Mar 24;22:320. doi: 10.1186/s12885-022-09357-y (PMC8953069; doi:10.1186/s12885-022-09357-y)
Supplement: Supplementary file 1 — Additional file 1. Supporting ResultsIC50 values, RNA-seq quality, optimal PCST networks and clustering and enrichment analysis of PCST generated networks are listed and figured in this additional file. [file 12885_2022_9357_MOESM1_ESM.docx]

**Context dependent isoform specific PI3K inhibition confers drug resistance in Hepatocellular carcinoma cells**

**Supplementary Results**

**IC_50_ values**

IC_50_ values at 72h calculated for both Sulforhodamine B (SRB) cytotoxicity assay and Real-time cell electronic sensing (RT-CES) system shown in Table 1.

**RNA-seq quality**

12 Single-end RNA reads, processed by Illimuna Genome Analyzer, were analyzed using FASTQC. According to FASTQC reports, length of 49bp single-end reads were well sequenced and no end bias was seen for the reads. An average of 33,465,904 single end 49bp clean reads were generated. The average mapping rate was 97.28%, resulting an average coverage of depth 41X (Table 2). Then, the mapped reads were counted using HTSeq-count given a set of genomic features. Library sizes were calculated through the gene set. Without any transformation on to counts, library sizes of genes were between 28-38 thousand.

**Optimal PCST Networks**

Forest-tuner was run for DEG results to find the best arrangements of parameters in this ranges; ω (1-10.0 or 5-15), β (1-15.0), μ (0.01-0.05). From the possible solutions, we selected the networks with the smallest mean degrees which were listed in Table 3. PCST networks were analyzed further through heatmap and Cytoscape visualizations.

**Clustering and enrichment analysis of PCST generated networks**

Cytoscape visualizations of inhibitor treated Huh7 and Mahlavu networks were presented in Figure 1 to 10. The networks were generated through PCST algorithm using Omics Integrator and sketched by Cytoscape tool. The nodes were colored by logFC values resulted from edgeR analysis and the darkness of the color increases by the increase of the value. Red and blue represented up- and downregulation respectively. Steiner nodes were shaped as diamond while input transcriptome nodes were figured as ellipse. Node size was directly correlated with betweenness centrality of nodes. Clusters were generated using betweenness centralities of the nodes using a community cluster algorithm (Glay). Clusters were boxed for a better representation. Then, the clusters were separately analyzed by BiNGO for gene set enrichment analysis and only selected significant Gene Ontology was added to the network through associated genes.

**Tables**

**Supplementary Table 1:** IC_50_ values at 72 hours of incubation, calculated based on SRB and RT-CES assays.

|  | SRB | | RT-CES | |
| --- | --- | --- | --- | --- |
| Inhibitors | Huh7 | Mahlavu | Huh7 | Mahlavu |
| Sorafenib | 8.0 μM | 6.6 μM | 10.0 μM | 10.0 μM |
| PIK-75 | ≤0.3μM | ≤0.3μM | 0.1 μM | 0.1 μM |
| TGX-221 | ≥40.0μM | ≥40.0μM | 10.0 μM | 15.0 μM |
| LY294002 | 3.8 μM | 8.7 μM | 10.0 μM | 10.0 μM |

**Supplementary Table 2:** Total sequences processed, map rate, average sequence length, GC% and coverage of the experiment.

|  | Total Sequences | Map Rate | Sequence Length | GC Content (%) | Coverage* |
| --- | --- | --- | --- | --- | --- |
| Akt Normal (Huh7) | | | | | |
| PIK-75 | 33192992 | 97.0 | 49 | 48 | 41 |
| TGX-221 | 32730853 | 97.3 | 49 | 50 | 40 |
| PIK-75 + Sorafenib | 34017394 | 97.4 | 49 | 49 | 42 |
| TGX-221 + Sorafenib | 28579376 | 97.6 | 49 | 49 | 35 |
| Sorafenib | 36506606 | 97.7 | 49 | 49 | 45 |
| DMSO | 37827459 | 97.5 | 49 | 48 | 47 |
| Akt Hyperactive (Mahlavu) | | | | | |
| PIK-75 | 34302640 | 96.0 | 49 | 47 | 42 |
| TGX-221 | 29776671 | 97.5 | 49 | 49 | 37 |
| PIK-75 + Sorafenib | 31897951 | 96.8 | 49 | 49 | 39 |
| TGX-221 + Sorafenib | 37221329 | 97.7 | 49 | 49 | 46 |
| Sorafenib | 32116151 | 97.4 | 49 | 50 | 39 |
| DMSO | 33421422 | 97.5 | 49 | 49 | 41 |
| ∗ Coverage = (Sequence Length x Total Sequences) / Human Transcriptome Length (39841315) | | | | | |

**Supplementary Table 3:** Selected parameters for PCST analysis using forest-tuner and number of nodes, terminals, and prizes of generated networks.

|  | ω | β | μ | (Terminal+ Steiner) Total Node | Prize Node | Mean Degrees |
| --- | --- | --- | --- | --- | --- | --- |
| Akt Normal (Huh7) | | | | | | |
| PIK-75 | 7.75 | 5.50 | 0.04 | (138+124) 262 | 171 | 24.83 |
| TGX-221 | 5.50 | 3.25 | 0.03 | (5+12) 17 | 5 | 23.08 |
| PIK-75 + Sorafenib | 10.0 | 10.0 | 0.02 | (145+101) 246 | 178 | 28.58 |
| TGX-221 + Sorafenib | 10.0 | 3.25 | 0.03 | (178+147) 325 | 213 | 23.86 |
| Sorafenib | 10.0 | 7.75 | 0.05 | (157+124) 281 | 187 | 27.19 |
| Akt Hyperactive (Mahlavu) | | | | | | |
| PIK-75 | 10.0 | 7.75 | 0.03 | (52+63) 115 | 84 | 29.14 |
| TGX-221 | 7.75 | 5.50 | 0.03 | (6+20) 26 | 6 | 16.70 |
| PIK-75 + Sorafenib | 10.0 | 3.25 | 0.01 | (321+236) 547 | 409 | 30.31 |
| TGX-221 + Sorafenib | 10.0 | 5.50 | 0.03 | (53+40) 93 | 75 | 34.65 |
| Sorafenib | 5.0 | 7.00 | 0.04 | (16+15) 31 | 27 | 19.67 |

**Figures**

Networks representing differential expression patterns in PI3K/AKT/mTOR and RAF/MEK/ERK signaling pathways in Huh7 and Mahlavu cell lines for differential inhibitory treatments are shown.


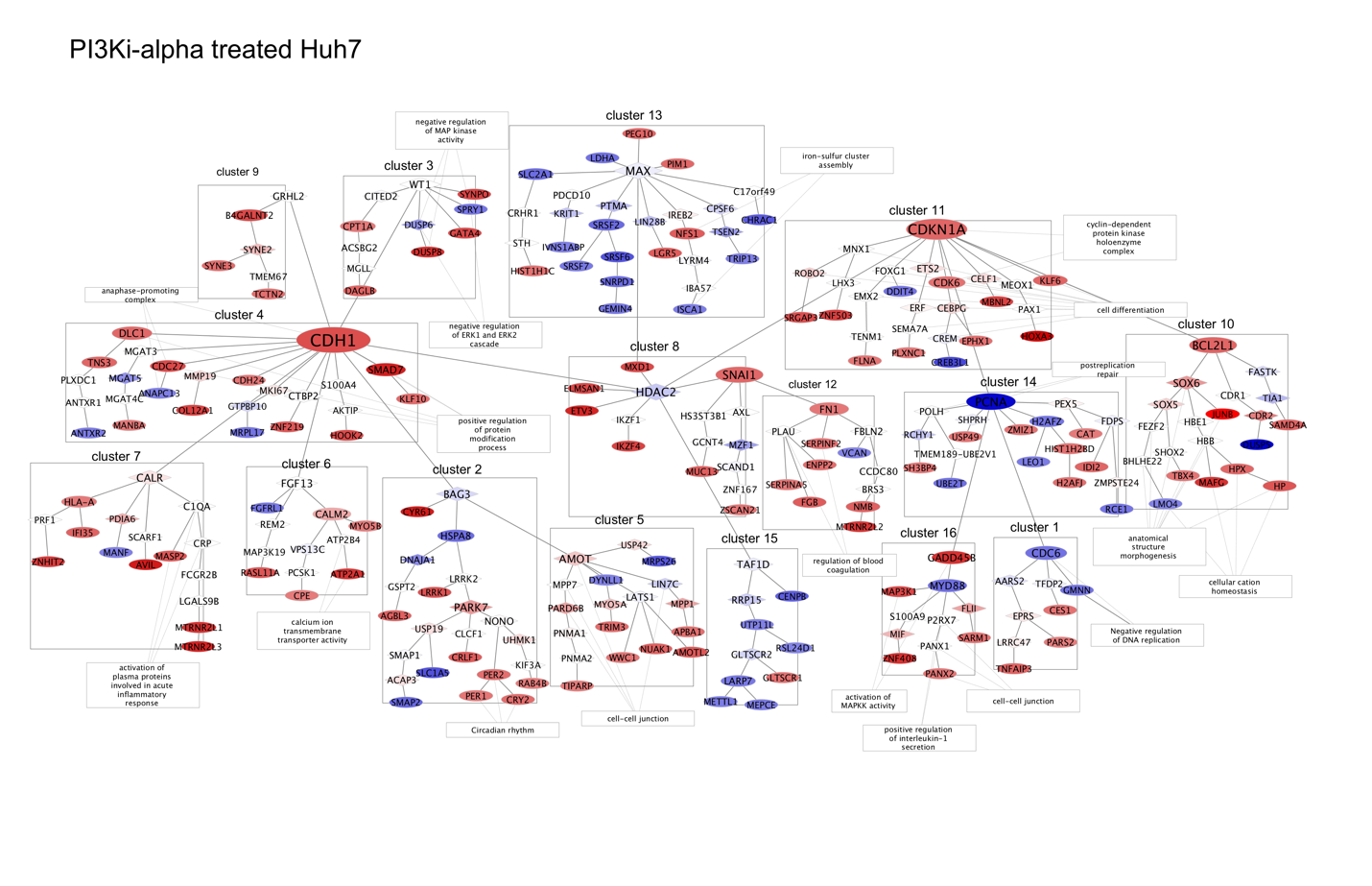


**Supplementary Figure 1: PIK-75 treated Huh7 cell line network**

**
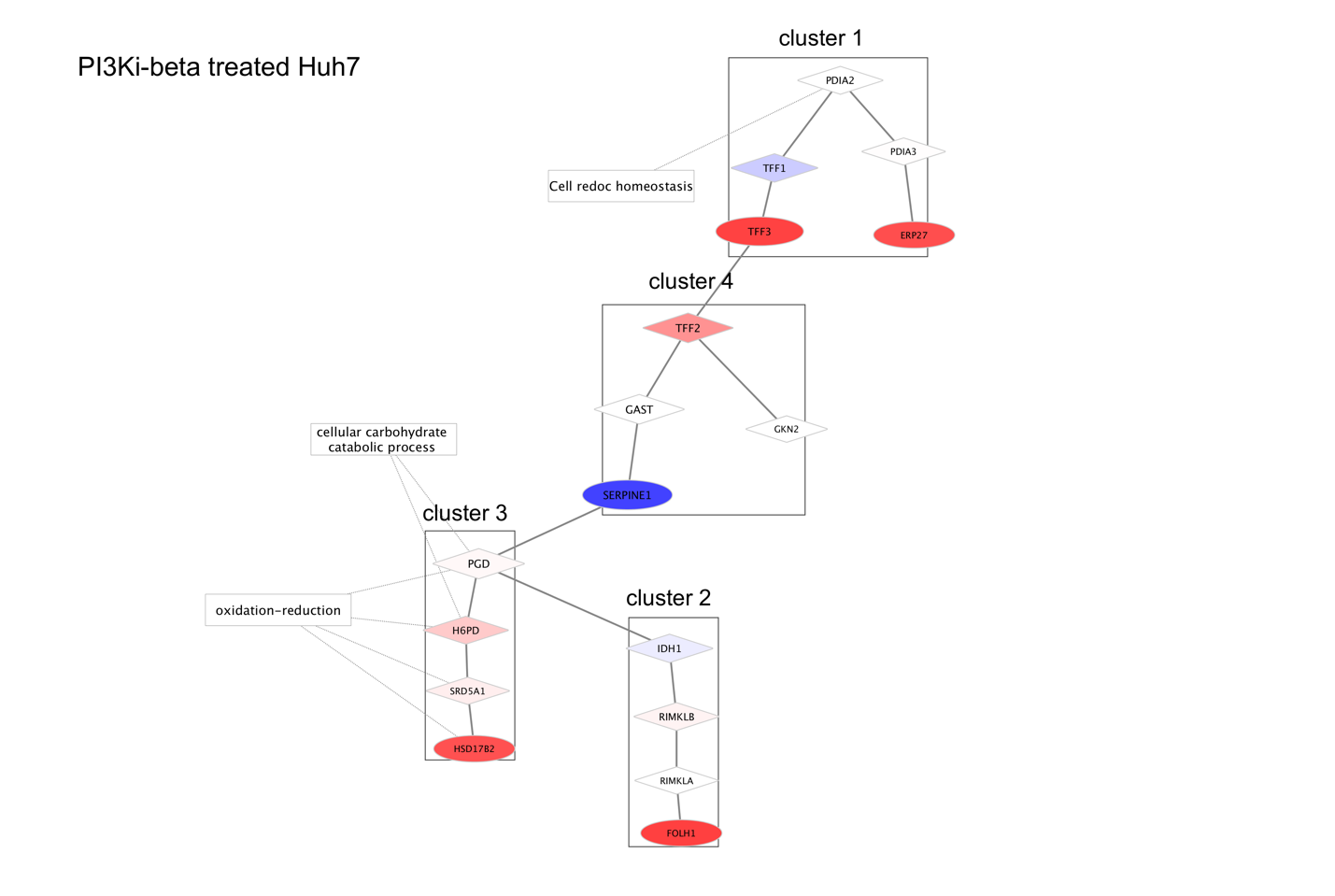
**

**Supplementary Figure 2: TGX-221 inhibitor treated Huh7 cell line network.**

**
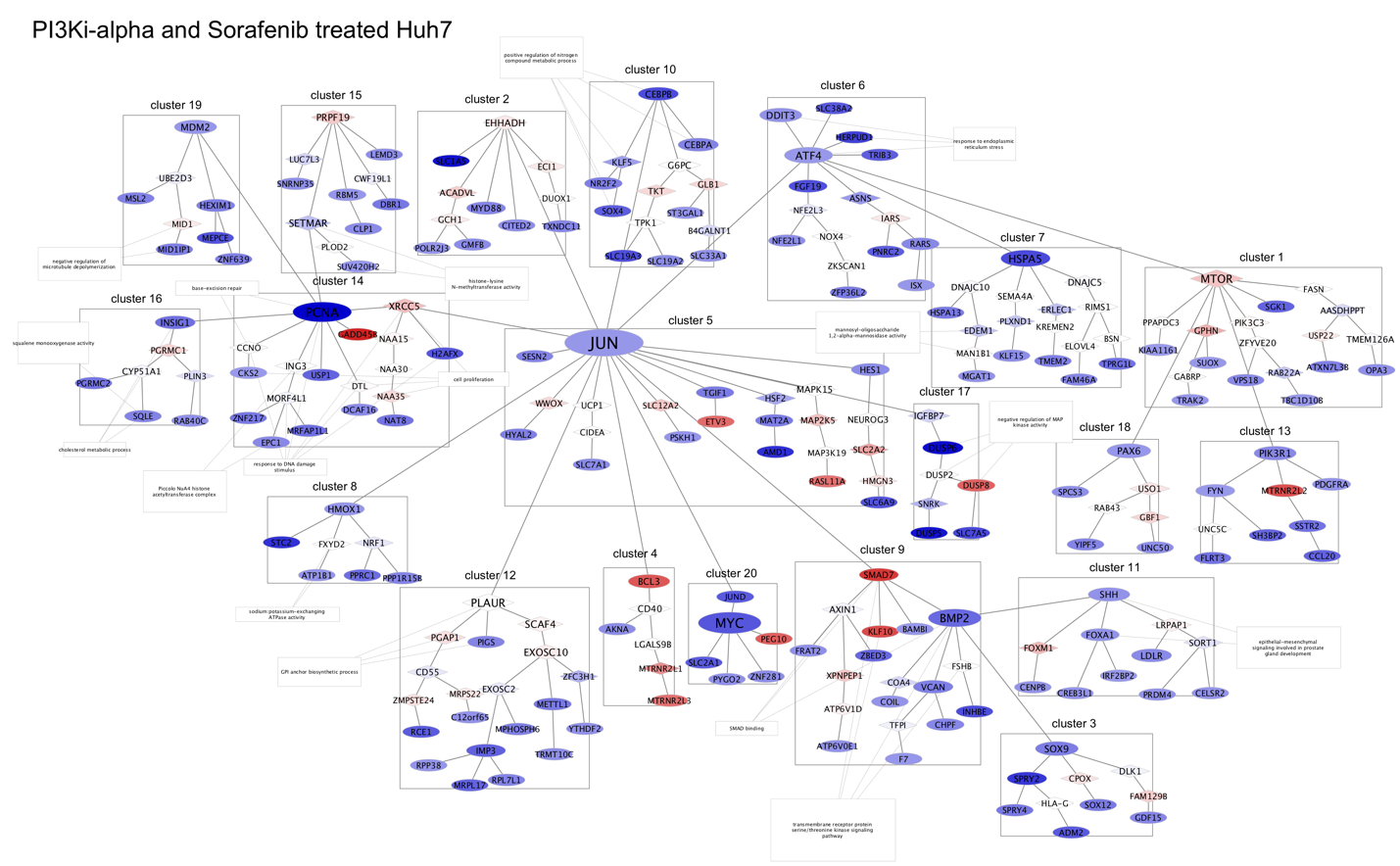
**

**Supplementary Figure 3: PIK-75 + Sorafenib treated Huh7 cell line network.**

**
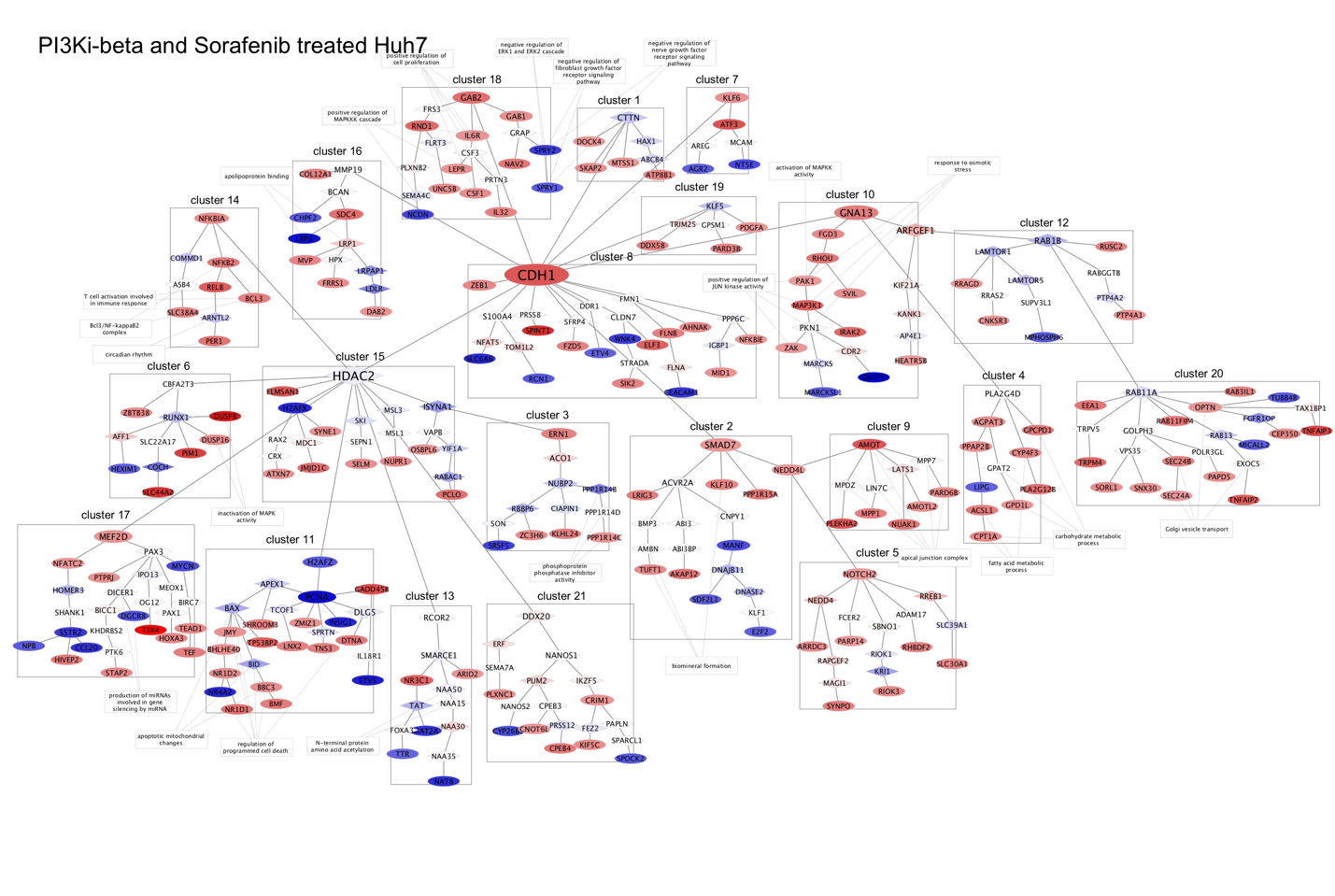
**

**Supplementary Figure 4: TGX-221 + Sorafenib treated Huh7 cell line network.**

**
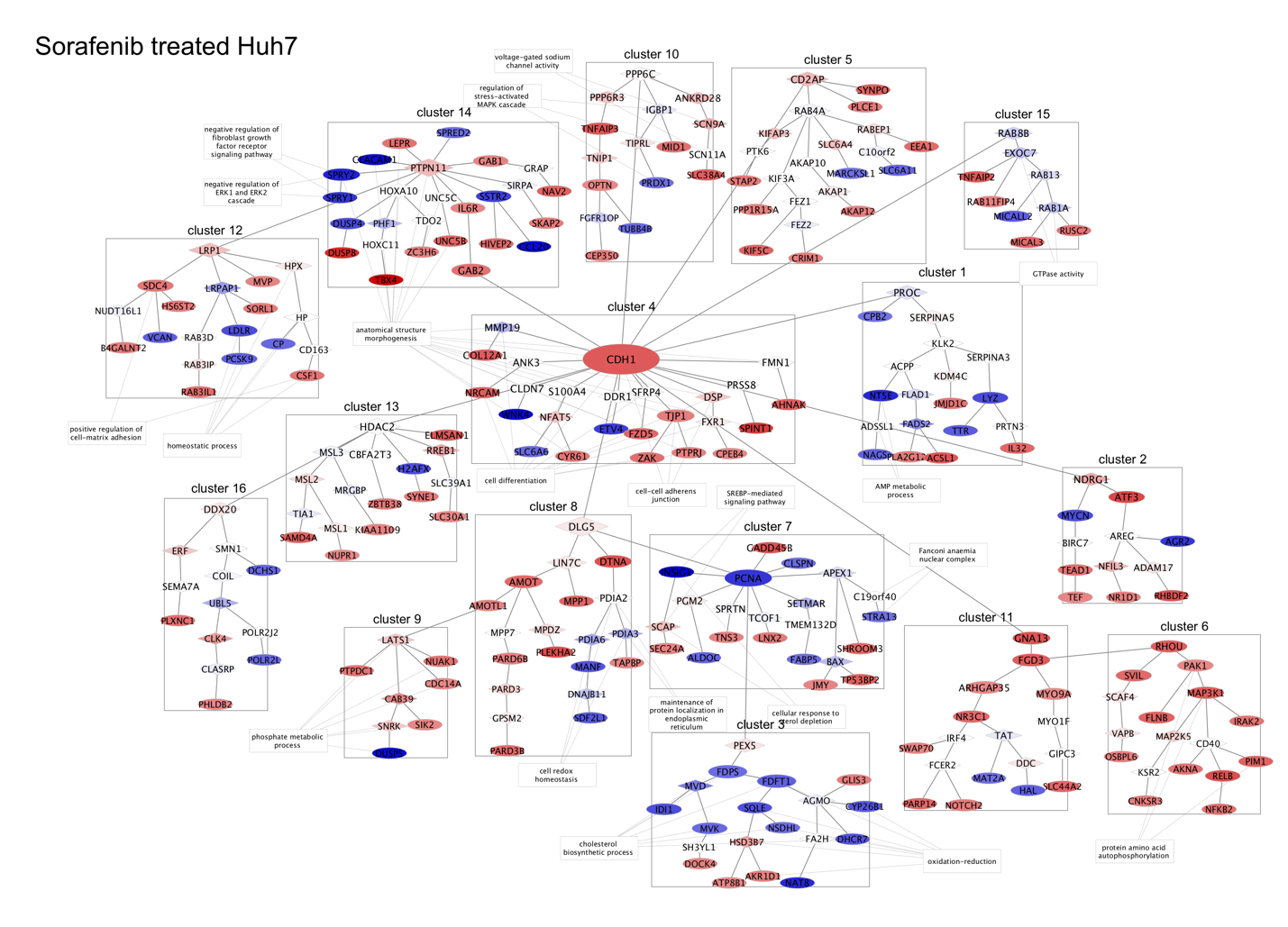
**

**Supplementary Figure 5: Sorafenib treated Huh7 cell line network.**

**
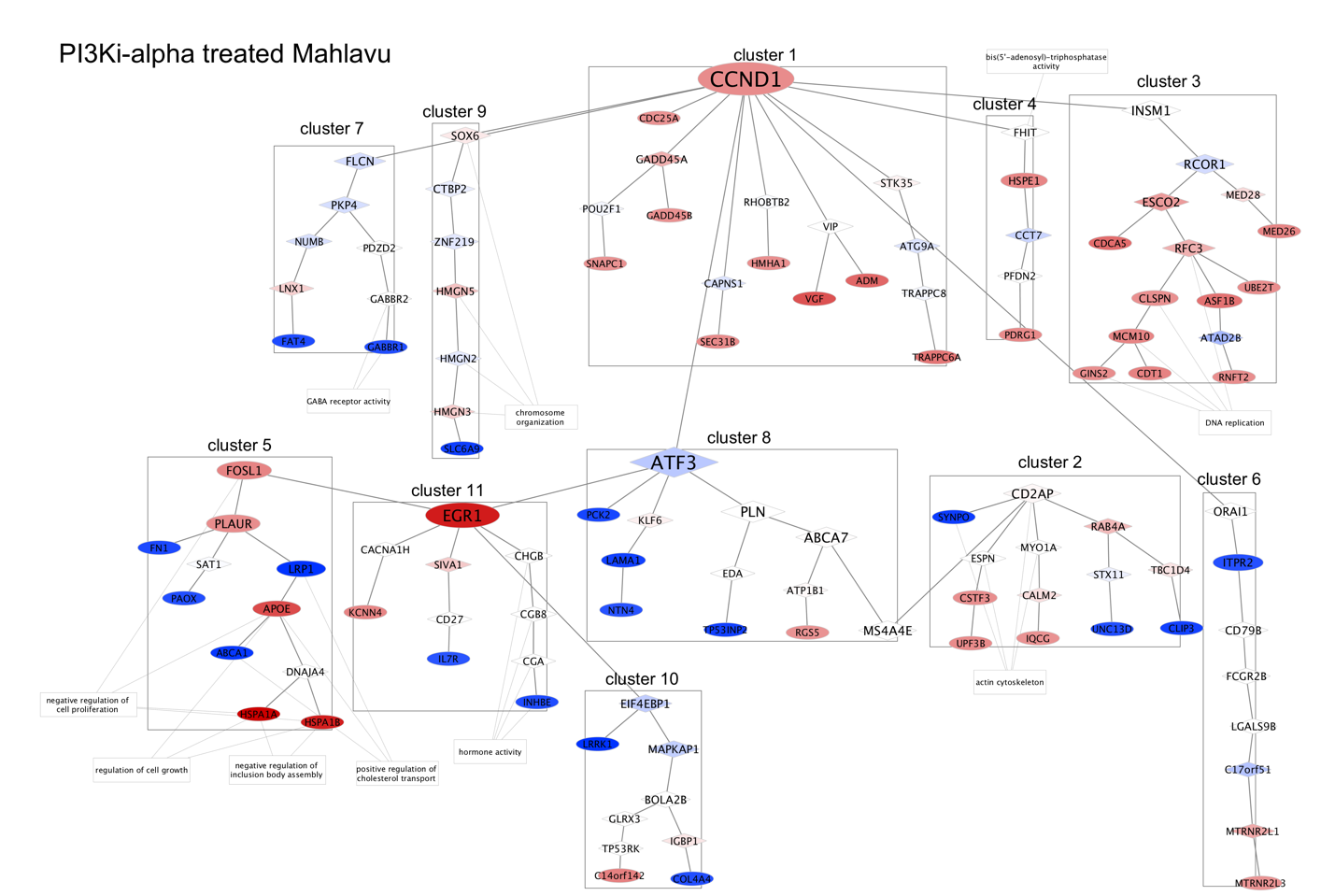
**

**Supplementary Figure 6: PIK-75 inhibitor treated Mahlavu cell line network.**

**
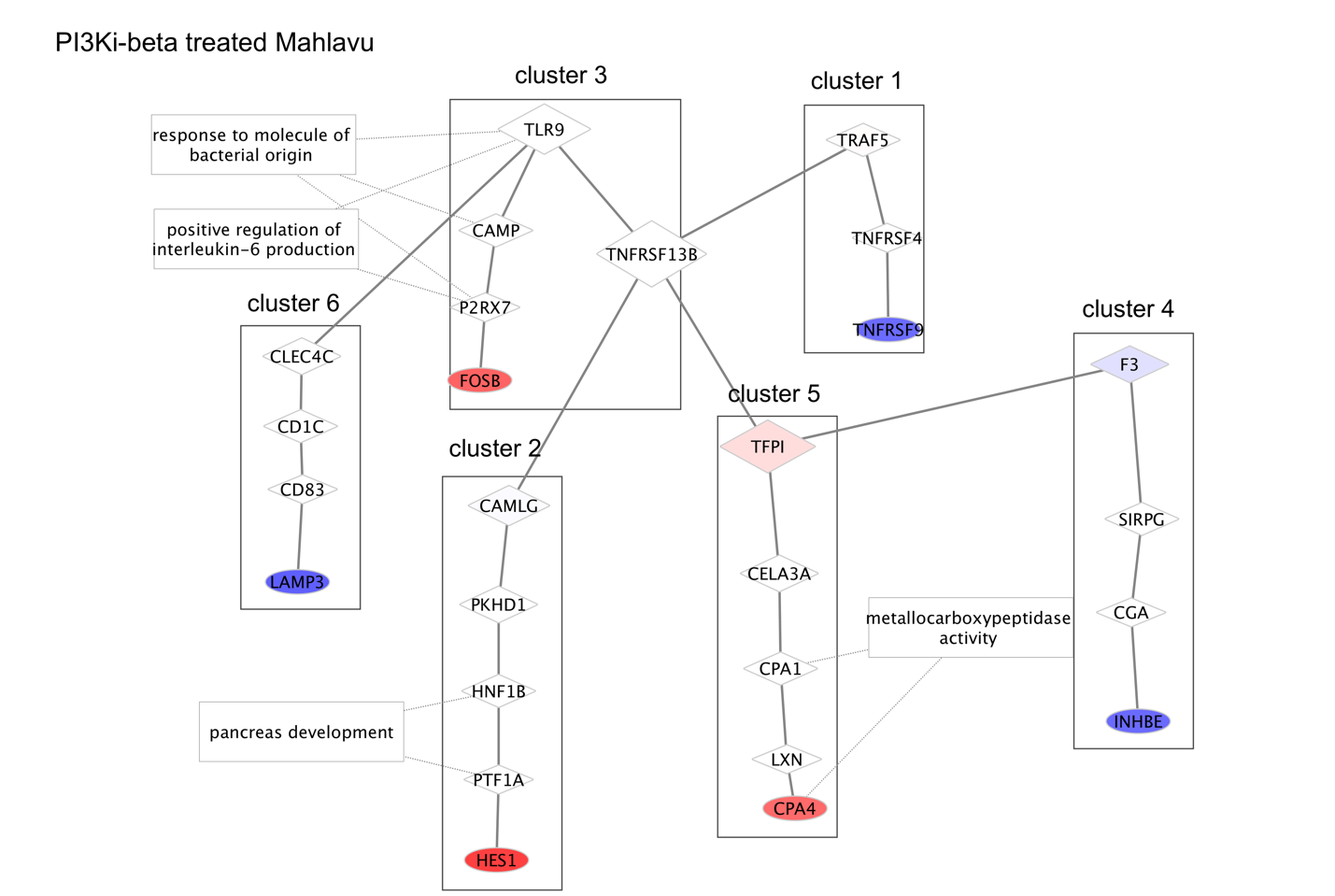
**

**Supplementary Figure 7: TGX-221 treated Mahlavu cell line network.**

**
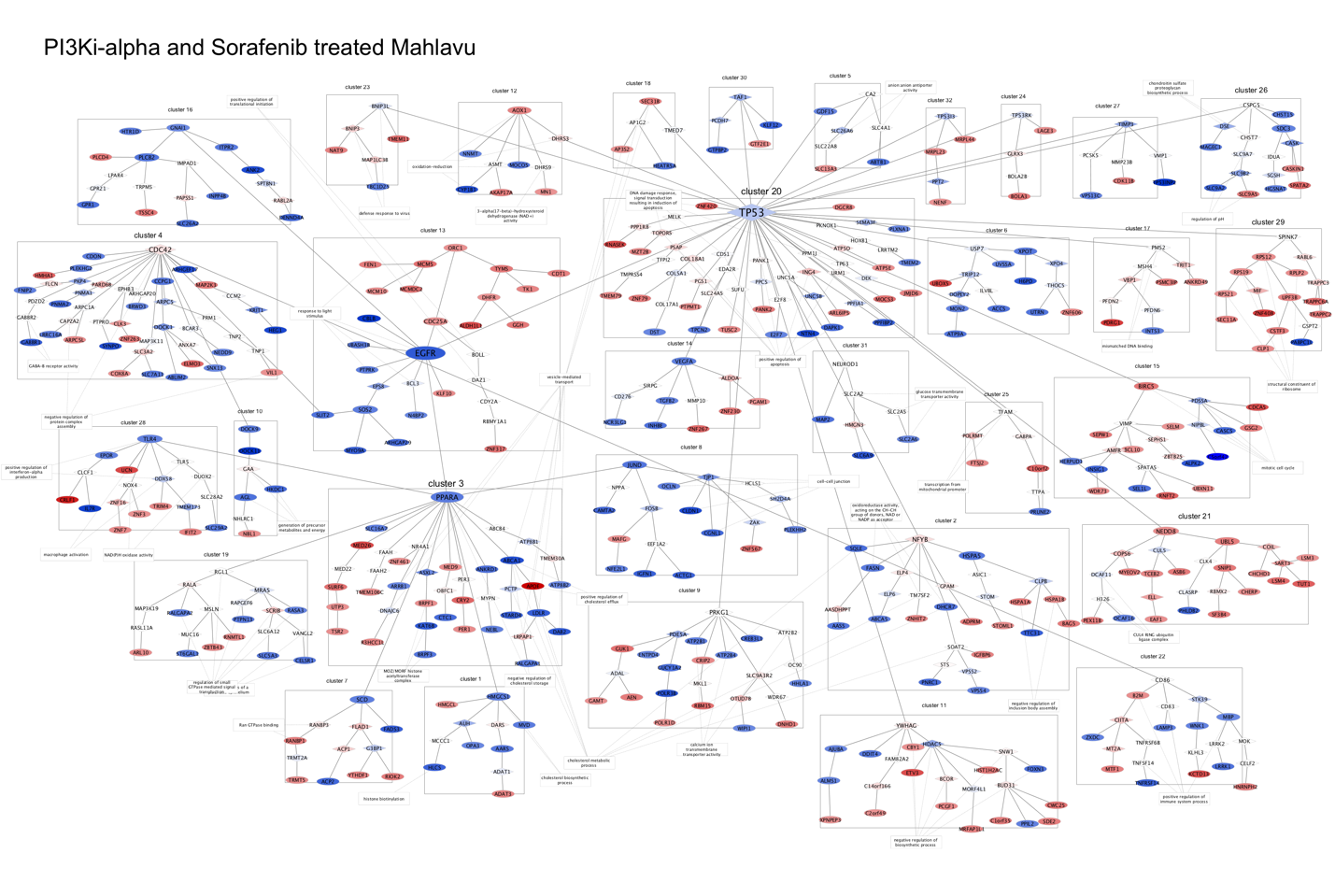
**

**Supplementary Figure 8: PIK-75 + Sorafenib treated Mahlavu cell line network.**

**
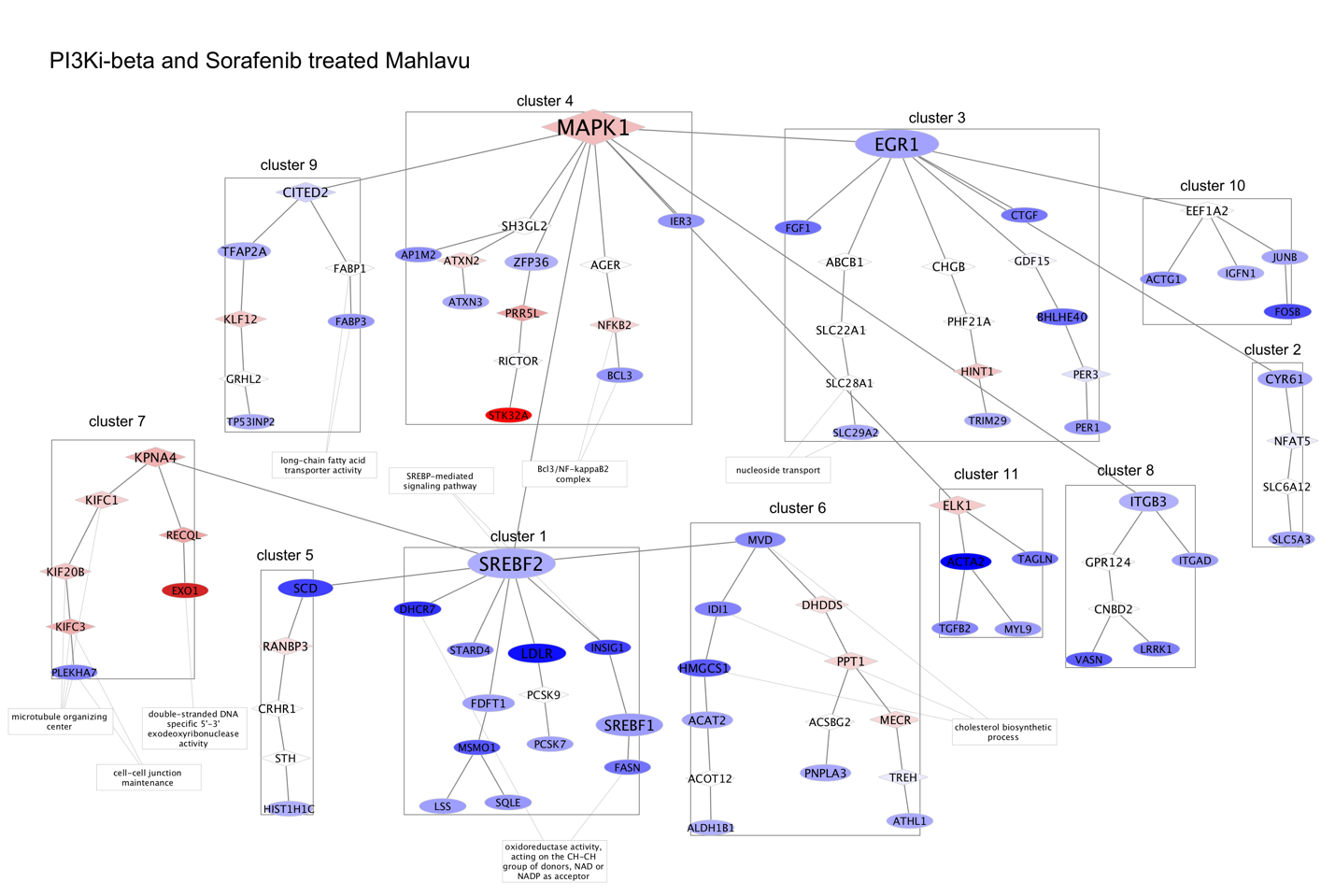
**

**Supplementary Figure 9: TGX-221 + Sorafenib treated Mahlavu cell line network.**

**
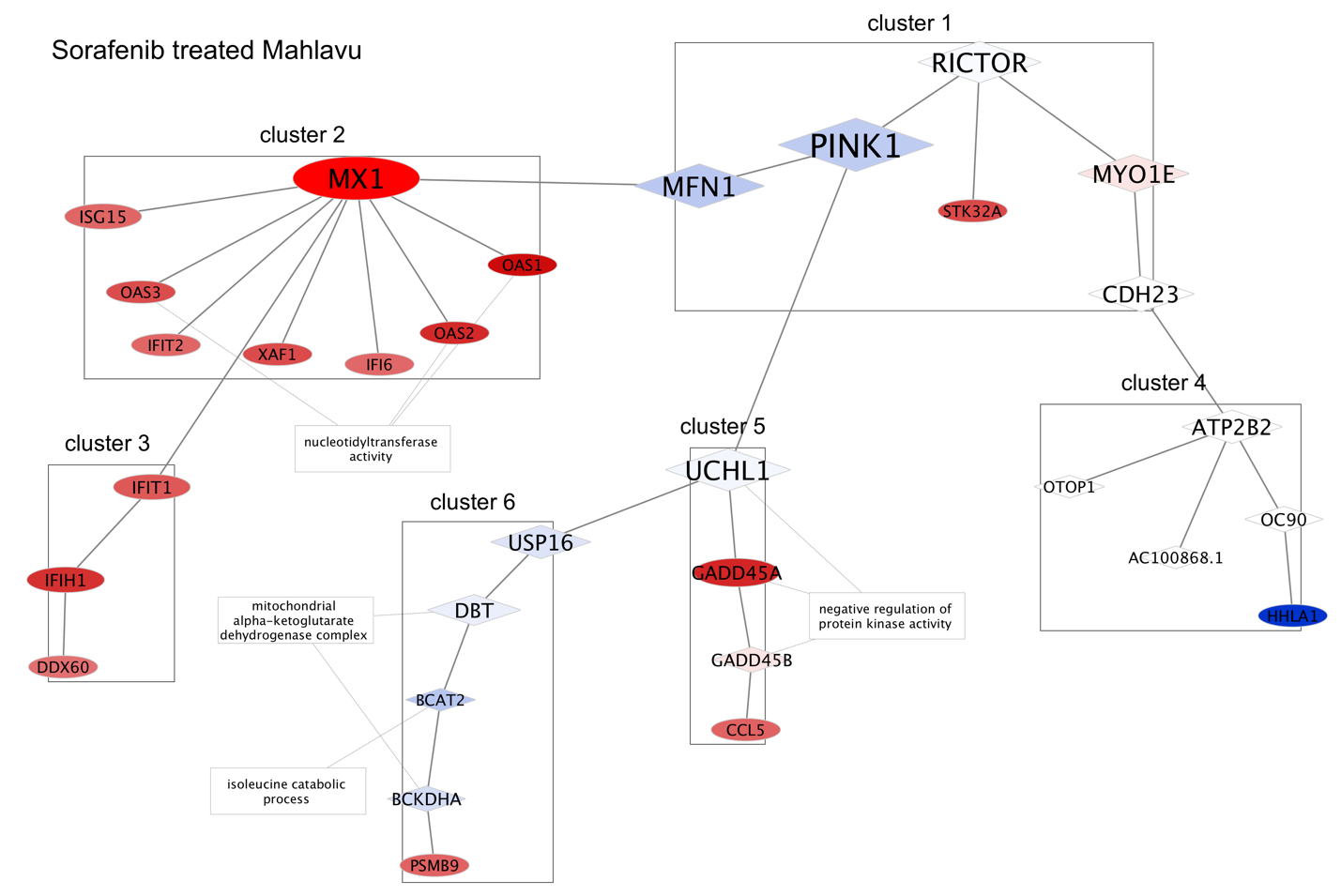
**

**Supplementary Figure 10: Sorafenib treated Mahlavu cell line network.**
